# High‐Intensity Exercise After Percutaneous Coronary Intervention in Previously Physically Active Patients: One‐Year Clinical Outcomes

**DOI:** 10.1111/sms.70194

**Published:** 2025-12-26

**Authors:** J. M. Guy, F. Schnell, S. Cade, S. Doutreleau, B. Gérardin, S. Armero, F. Chagué, C. Hédon, S. Guérard, F. Ivanes, L. Chevalier

**Affiliations:** ^1^ Cardio‐Respiratory Rehabilitation Centre Saint‐Priest‐en‐Jarez France; ^2^ Department of Sports Medicine University Hospital Pontchaillou Rennes France; ^3^ LTSI, INSERM, U1099 University of Rennes Rennes France; ^4^ CIC 1414, INSERM, University Hospital Pontchaillou University of Rennes Rennes France; ^5^ Department of Cardiology Clinique du Millénaire Montpellier France; ^6^ TIMC Laboratory, Grenoble Alpes University Grenoble France; ^7^ Hôpital Marie Lannelongue, Groupe Hospitalier Paris Saint Joseph University of Paris‐Saclay Plessis‐Robinson France; ^8^ Department of Cardiology Hôpital Européen Marseille Marseille France; ^9^ Department of Cardiology Dijon Bourgogne University Hospital Dijon France; ^10^ PhyMedExp, University of Montpellier, INSERM, CNRS, Cardiology and Physiology Department University Hospital Montpellier Montpellier France; ^11^ Private Cardiology Practice Bron France; ^12^ Department of Cardiology Tours University Hospital Tours France; ^13^ UMR1327 ISCHEMIA Membrane Signalling and Inflammation in Reperfusion Injuries University of Tours Tours France; ^14^ Clinique du Sport Bordeaux‐Mérignac France

**Keywords:** coronary artery disease, exercise, percutaneous coronary intervention, return to play, stent, veteran athlete

## Abstract

The benefits of regular physical activity are well demonstrated in secondary cardiovascular prevention, but data on high‐intensity exercise after percutaneous coronary intervention (PCI) remain limited. This study aimed to assess whether resuming high‐intensity exercise within the first year after PCI was associated with an increased risk of cardiovascular (CV) events. We prospectively followed 1154 patients who had undergone PCI without prior revascularization. All patients engaged in regular exercise prior to PCI. We compared the 1‐year outcome of patients who resumed high‐intensity exercise with the remaining patients. Within the first year 91.6% of patients resumed exercise (18.0% high‐intensity, 41.0% moderate‐intensity, 32.6% light‐intensity), predominantly endurance‐based (93.3%). No CV death was reported, atrial fibrillation occurred in 27 patients (2.3%), ischemic stroke in 4 (0.3%), ventricular arrhythmias in 9 (0.8%), acute heart failure in 8 (0.7%), and new coronary event in 30 (2.6%); including 21 new stenoses, 4 in‐stent restenoses, and 5 stent thromboses. Four patients experienced an acute coronary syndrome, including 2 during exercise. The 205 patients in the high‐intensity exercise group reported a higher training load (7.0 [5.0–8.0] hours/week vs. 4.0 [2.0–6.0], *p* < 0.0001). The incidence of CV events did not differ significantly between the groups (total CV events: 5.9% vs. 7.0%, *p* = 0.541; new coronary events: 1.5% vs. 2.9%, *p* = 0.250 respectively in the high‐intensity exercise group vs. the remaining patients). These findings indicate no observed increase in short‐term CV events among previously active patients resuming high‐intensity exercise after PCI, but further studies are required to determine whether these observations are generalisable.

## 1 Introduction

Given the clear benefits of regular moderate‐intensity physical activity on morbidity and mortality in both primary and secondary cardiovascular prevention [1, 2, 3, 4], current guidelines emphasize the importance of cardiac rehabilitation and encourage coronary artery disease (CAD) patients to resume regular physical activity [5]. But there is also a transient increase in the risk of sudden cardiac arrest during vigorous exercise due to CAD [6, 7, 8] related to plaque rupture or demand ischemia. Some studies suggest that high‐intensity exercise may contribute to the development of coronary atherosclerosis [9, 10, 11]; moreover, it might expose the patients to the transient pro‐thrombotic and pro‐inflammatory effects induced by physical exertion and dehydration [12, 13], as well as increased shear stress on a recently stented artery or vulnerable untreated plaques prone to rupture [14]. European and North American guidelines, mostly based on expert consensus, have been established to codify the resumption of more intense unsupervised exercise, including competitive sports, following percutaneous coronary intervention (PCI) [15, 16]. However, there is limited data on the outcome of high‐intensity exercise in the initial phase after PCI. Our group published a retrospective study in 2019 [17] on a modest sample of 108 CAD patients, reporting an incidence of severe coronary events of approximately 16%, particularly among those with bare‐metal stents. Given the almost exclusive use of drug‐eluting stents in recent years, a prospective study with a larger sample size was needed to more accurately describe the type and frequency of cardiovascular (CV) complications that may occur when previously active patients resume high‐intensity exercise. The objective of our study was therefore to prospectively follow‐up (FU) a cohort of CAD patients engaged in regular exercise and to determine whether resuming high‐intensity exercise was associated with the occurrence of a subsequent coronary event within the first year following their initial PCI.

## 2 Material and Methods

### 2.1 Study Population

**FIGURE 1:**
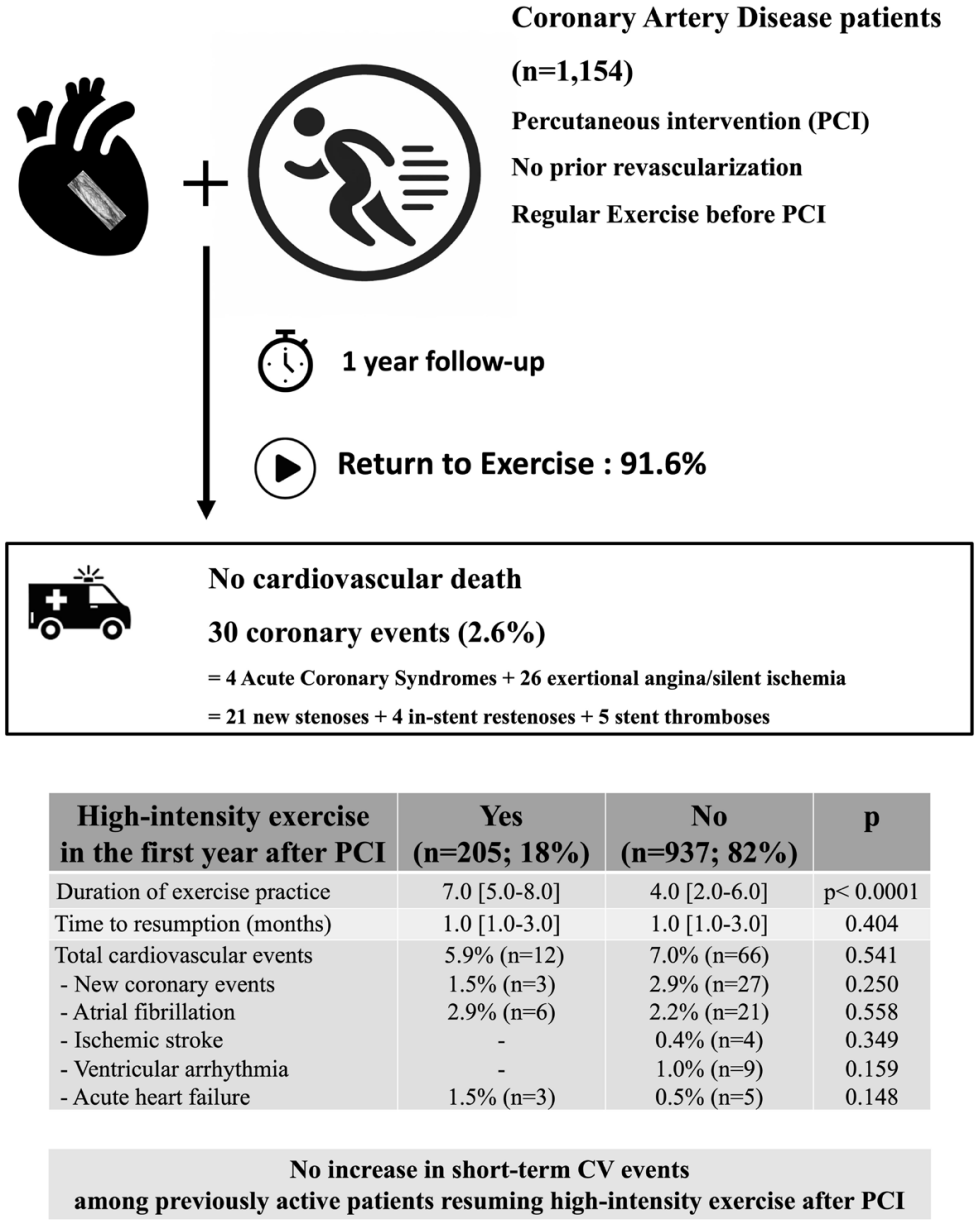
Graphical abstract summarizing the study rationale and main findings.

This was a prospective multicentre French study involving 37 cardiology centres. Our study population included 1262 patients who had undergone PCI without a prior history of revascularization procedures. They had to be enrolled during the hospitalization for PCI, whether performed in the context of an acute or chronic coronary syndrome. To qualify for inclusion, patients had to engage in regular exercise (Figure 1). Of the initial cohort, 108 patients (8.6%) were lost to follow‐up, and 1154 completed the 1‐year follow‐up.

### 2.2 Study Protocol

We recorded the number of hours of exercise, the sport discipline (classified as skill, power, mixed, and endurance exercise) [15], its intensity, and participation in competitions. Exercise intensity was categorized into three levels based on the Borg Rating of Perceived Exertion (RPE; scale 6–20) [18] and the Talk Test (TT) [19, 20]: light‐intensity exercise, defined as exercise without shortness of breath and allowing easy conversation (Borg RPE: 9–11); moderate‐intensity exercise, involving physical exertion with moderate shortness of breath (Borg RPE: 12–14); and high‐intensity exercise, causing marked shortness of breath, with conversation limited to a few words (Borg RPE ≥ 15) [21]. Importantly, in our cohort, participation in competitions did not necessarily reflect high‐intensity exercise (e.g., competitive golf).

The choice of treatments and FU assessments were left to the discretion of the cardiologists in charge of the patients. The FU period lasted 12 months from the date of the initial PCI. All CV events were recorded through a standardized phone interview conducted by a single operator, using a structured questionnaire. Patients were asked to provide medical reports related to any potential events. If neither the patient nor their relatives responded, the cardiologist who had initially included them was contacted; if he had no information as regards the patient, we considered the patient as lost to FU and consulted the French government database of deceased persons (www.deces‐en‐france.fr). The CV events of interest included CV‐related mortality and hospitalizations for CV reasons, particularly coronary events such as new stenosis, in‐stent restenosis, or stent thrombosis. During FU, the number of hours of exercise, its intensity, discipline and participation in competitions were reassessed using the same classification as at inclusion by a single operator, with an additional category for patients who had stopped all exercise.

The study was approved by the Dijon Committee for the Protection of Persons (approval no. 2018‐A01169‐46) and received a compliance agreement from the French National Commission on Informatics and Liberties (CNIL) on April 26, 2018 (no. 2177339 v0). The study was conducted in accordance with the Declaration of Helsinki, and all participants provided informed consent.

### 2.3 Statistical Analysis

The normality of continuous variables was assessed using a Shapiro–Wilk test, and could not be assumed for any variable. Categorial variables were expressed as percentages, and continuous variables as median with interquartile range (IQR). Comparisons were performed between patients with complete FU and those lost to FU, patients with and without subsequent coronary events, and between patients who did or did not engage in high‐intensity exercise during FU. The Mann–Whitney was used for comparisons of continuous variables, and the Chi^2^ test for categorial variables. Comparisons of continuous variables before and after PCI were performed using a Wilcoxon test. Tests with *p*‐values less than 0.05 were considered statistically significant. All Statistical analyses were performed using SPSS (v.20 SPSS Inc.; Chicago, IL).

## 3 Results

A total of 1262 patients were included in the study. However, 108 patients (8.6%) were lost to FU; none of them died during the study period. The group of patients lost to FU was comparable to the rest of the population in terms of age, gender, duration of exercise at inclusion, indication for PCI, left ventricular ejection fraction (LVEF), and the presence of LV wall motion abnormality on initial echocardiography (Table 1).

**TABLE 1:** Comparison of the “lost‐to‐follow‐up” group vs the population followed for 1 year.

	Global population (*n* = 1262)	Patients Lost‐to‐FU (*n* = 108)	Population 1‐year FU (*n* = 1154)	*p*
Age (years)	60.0 [53.0–68.0]	58.5 [51.0–69.0]	61.0 [53.0–68.0]	0.274
Male gender	88.5%	84.9%	88.8%	0.227
Duration of exercise (h/week)	4.0 [3.0–7.0]	4.0 [3.0–6.9]	4.0 [3.0–7.0]	0.777
Emergency coronary angiography	74.2%	76.2%	74.0%	0.624
LVEF (%)	60.0 [50–61]	60.0 [50.0–60.0]	60.0 [50–61]	0.502
LV wall motion abnormality	43.9%	45.5%	43.8%	0.735

Abbreviations: FU, follow‐up; LV, left ventricular; LVEF, left ventricular ejection fraction.

### 3.1 General Characteristics, Interventional Procedures, Pharmacological Treatments and Exercise of the Study Population

Among the 1154 patients who completed the 1‐year FU, the vast majority (88.8%) were men, with a median age of 61.0 years [53.0–68.0]. The main CV risk factors were hypercholesterolemia (41.7%) and arterial hypertension (37.8%), followed by active smoking (25.1%), obesity (11.9%), and diabetes mellitus (10.6%). Only 25.3% of the population were free of these traditional risk factors (Table 2). At the time of CAD diagnosis, the median number of hours of exercise per week among the population was 4.0 [3.0–7.0], with 36.3% of the population engaging in at least 6 h of exercise per week. Regarding exercise, 28.2% of patients engaged in light‐intensity exercise, 45.5% in moderate‐intensity exercise, and 26.3% in high‐intensity exercise; 12.1% of individuals participated in competitive sports. Most subjects engaged in endurance exercise before PCI (89.5%), either exclusively (78.9%) or in combination with mixed (5.5%), skill (2.9%), or power (2.0%) exercise. An additional 5.6% practiced only mixed exercise, 2.5% only skill‐based exercise, and 1.7% only power‐based exercises. The remaining patients (1.0%) participated in other, less common combinations of exercise types. Urgent coronary angiography was performed in 74.0% of cases; the median number of stents implanted per patient was 1.0 [1.0–2.0]. Patients predominantly received drug‐eluting stents, with only 0.6% receiving at least one bare‐metal stent. The majority of patients (76.3%) had a single‐vessel disease. The stenting distribution was as follows: left anterior descending coronary artery (60.7%), right coronary artery (37.7%), circumflex coronary artery (25.8%), and left main coronary artery (2.4%). A complete revascularization was achieved in 78.6% of patients from the outset. On the initial echocardiography, median LVEF was 60.0% [50.0–61.0], with wall motion abnormality in 43.8% of cases. Regarding pharmacological treatments following PCI, all patients were on low‐dose aspirin, 62.1% on ticagrelor, 30.3% on clopidogrel, 5% on prasugrel, and 3.1% on oral anticoagulants. Statins were prescribed in 93.4% of patients, beta‐blockers in 74.2%, calcium channel blockers in 8.3%, and angiotensin‐converting enzyme inhibitors (ACEIs) or angiotensin II receptor blockers (ARBs) in 67.6%.

**TABLE 2:** Characteristics of the overall population and comparison of the groups with and without coronary events (new stenosis, thrombosis, in‐stent restenosis).

	Population 1‐year FU (*n* = 1154; 97.4%)	Population without coronary event (*n* = 1124)	Population with coronary event (*n* = 30; 2.6%)	*p*
Age at inclusion	61.0 [53.0–68.0]	61.0 [53.0–68.0]	60.5 [56.3–65.5]	0.904
Men	88.8%	88.8%	90.0%	0.836
Diabetes mellitus at inclusion	10.6%	10.5%	13.3%	0.619
Smoking at inclusion	25.1%	25.3%	16.7%	0.280
Smoking at 1‐year FU	7.1%	7.2%	3.6%	0.459
Obesity at inclusion	11.9%	12.1%	3.3%	0.143
Arterial hypertension at inclusion	37.8%	37.8%	36.7%	0.898
Hypercholesterolemia at inclusion	41.7%	41.7%	43.3%	0.858
None of those CV risk factor at inclusion	25.3%	25.4%	23.3%	0.799
Emergency coronary angiography	74.0%	74.1%	70.0%	0.612
LV wall motion abnormality	43.8%	44.1%	33.3%	0.267
LVEF at inclusion (%)	60.0 [50.0–61.0]	60.0 [50.0–61.0]	60.0 [50.0–65.5]	0.445
Complete revascularization	78.6%	78.7%	72.4%	0.413
Number of stents per patient	1.0 [1.0–2.0]	1.0 [1.0–2.0]	1.0 [1.0–2.0]	0.840
Single‐vessel disease	76.3%	76.3%	79.3%	0.702
Patient stented on LMCA	2.4%	2.3%	6.7%	0.126
Patient stented on LAD	60.7%	60.6%	66.7%	0.501
Patient stented on CX	25.8%	26.1%	16.7%	0.246
Patient stented on RCA	37.7%	37.8%	33.3%	0.617
Drug‐eluting stents	99.4%	99.4%	100%	0.665
*Exercise*				
Duration of exercise before PCI (hours/week)	4.0 [3.0–7.0]	4.0 [3.0–7.0]	4.0 [3.0–8.0]	0.478
Endurance exercise before PCI	89.5%	89.5%	90.0%	0.932
Intensity of exercise before PCI				0.512
Light‐intensity	28.2%	28.5%	20.0%	
Moderate‐intensity	45.5%	45.5%	46.7%	
Hight‐intensity	26.3%	26.1%	33.3%	
Competition before PCI	12.1%	12%	13.8%	0.775
Duration of exercise after PCI (hours/week)	5.0 [3.0–7.0]	5.0 [3.0–7.0]	4.0 [2.0–5.3]	0.042
Duration of exercise after PCI (only patient who resumed exercise, hours/week)	5.0 [3.0–8.0]	5.0 [3.0–8.0]	4.0 [3.0–6.0]	0.137
Endurance exercise after PCI	93.3%	93.2%	96.0%	0.580
Intensity of exercise after PCI				0.283
No exercise	8.5%	8.3%	16.7%	
Light‐intensity	32.6%	32.5%	36.7%	
Moderate‐intensity	41.0%	41.1%	36.7%	
Hight‐intensity	18.0	18.2%	10%	
Competition after PCI	4.9%	5.1%	0	0.205
Change in intensity of exercise (post‐pre PCI)				0.020
Decrease	28.9%	28.3%	50%	
No change	64.5%	64.9%	50%	
Increase	6.6%	6.7%	0	
Time to resumption (months)	1.0 [1.0–3.0]	1.0 [1.0–3.0]	2.0 [1.0–3.0]	0.250

Abbreviations: CV, cardio‐vascular; CX, circumflex coronary artery; FU, Follow‐up; LAD, left anterior descending coronary artery; LMCA, left main coronary artery; LV, left ventricular; LVEF, left ventricular ejection fraction; PCI, percutaneous coronary intervention; RCA, right coronary artery.

### 3.2 One Year‐FU Data: Patients Characteristics, Treatments, Cardiovascular Events and Exercise

At 1 year, 97.2% of patients reported still receiving antiplatelet therapy, with 66.2% on monotherapy and 31.0% on dual therapy; 6.5% were on anticoagulant therapy. The use of statins remained stable (92.1%), with the introduction of Ezetimibe in 38.5% of subjects; 72.2% of patients were on beta‐blockers and 12.7% on calcium channel blockers, while 69.5% were on ACEIs or ARBs. In the group that experienced a new coronary event, no patient reported improper adherence to antiplatelet therapy. Regarding smoking, it was continued by 7.1% of subjects.

No CV‐deaths were reported at 1 year. Three male patients died during FU (0.3%), but their deaths were unrelated to their initial CAD (severe COVID‐19 infection, digestive cancer, and cerebral hemorrhage). From a cardiovascular perspective, atrial fibrillation occurred in 27 patients (2.3%), ischemic stroke in 4 (0.3%), ventricular arrhythmias in 9 (0.8%), and acute heart failure in 8 (0.7%). Regarding coronary outcomes, 30 patients (2.6%) showed disease progression. These events affected both men and women (2.3% of women and 2.6% of men, *p* = 0.836). Four patients (0.3%) experienced an ACS, including one during low‐intensity exercise (walking, *n* = 1) and one during moderate‐intensity exercise (a 50 km cycling session on flat terrain at a speed < 20 km/h, *n* = 1), while the other 2 events occurred at rest. The other 26 patients (2.3%) presented with recurrent exertional angina or silent ischemia detected by a non‐invasive ischemic test. The majority of those patients (*n* = 21; 1.8%) experienced disease progression at a new site; the remaining 9 patients (0.8%) had an event at the site of the initial angioplasty with in‐stent restenosis (*n* = 4; 0.3%) or stent thrombosis (*n* = 5; 0.4%); all cases of stent thromboses were late thromboses, occurring between 6 months and 1 year.

Regarding exercise, information was unavailable for 12 patients; none of them experienced new CV or coronary event. At 1 year, 8.5% of patients had not resumed exercise, 32.6% engaged in light‐intensity exercise, 41.0% in moderate‐intensity exercise, and 18% in high‐intensity exercise, with 4.9% participating in competitive sports. Most subjects engaged in endurance exercise after PCI (93.3%), either exclusively (85.7%) or in combination with mixed (3.2%), skill (2.2%), or power (2.0%) exercise. An additional 3.1% practiced only mixed exercises, 1.8% only skill‐based exercise, and 0.7% only power‐based exercise. The remaining patients (1.3%) participated in other, less common combinations of exercise types. When analyzing changes in the intensity of their exercise compared to the pre‐PCI period, 64.5% of subjects maintained the same intensity level, 28.9% reduced their intensity, whereas only 6.6% increased it. In terms of training volume, the average duration of exercise significantly decreased after PCI, primarily driven by a reduction in extreme training hours, as confirmed by the Wilcoxon test, which indicates a significant reduction in hours of practice with a shift in rank distributions (negative ranks: 45.4%, positive ranks: 34.2%, ties: 20.4%, *p* < 0.0001). However, this change was not reflected in the median and interquartile range values (4.0 [3.0–7.0] vs. 5.0 [3.0–7.0], respectively before and after the procedure). Exercise was resumed after a median time of 1.0 month [1.0–3.0], with 78.5% of patients having resumed them by 3 months.

The characteristics of patients with subsequent coronary events (*n* = 30) and those without events (*n* = 1124) did not differ in terms of age, CV risk factors, initial clinical status, baseline exercise level, initial stented arteries (Table 2). Patients in the subsequent coronary event group engaged in fewer hours of exercise (4.0 [2.0–5.3] vs. 5.0 [3.0–7.0], respectively, *p* = 0.042). However, this difference was no longer significant when excluding patients who had completely stopped exercise. There was no difference in the intensity of exercise before or after PCI, nor in competitive sports participation. However, a significant difference emerged between the 2 groups when examining changes in exercise over time. In the subsequent coronary event group, the patients were more likely to have reduced their exercise intensity. Indeed, the proportion of patients who decreased their exercise intensity was higher in the new event group (50% vs. 28.3%), while those maintaining stable exercise levels were lower (50% vs. 64.9%), and none had increased their exercise intensity compared to the no‐event group (0% vs. 6.7%); this difference was statistically significant (*p* = 0.020).

The patients who resumed high‐intensity exercise within the first year after PCI (*n* = 205, 18%) were younger, more likely to be male, had fewer CV risk factors at baseline than the other patients (Table 3). In fact, they were more often free of traditional CV risk factors and less frequently diabetic or smokers at the time of inclusion. There were no differences between groups as regards to LVEF, presence of LV wall motion abnormalities, urgent PCI, complete revascularization, single‐vessel disease, number of stents per patient, or the use of drug‐eluting stents. As regards to baseline exercise, those who returned to high‐intensity exercise after PCI were more often involved in competitive sport, had practiced more hours of exercise per week and at higher intensity before PCI than the remaining patients. The timing of resumption did not differ between both groups, but those who resumed high‐intensity exercise after PCI engaged in more hours of exercise per week post‐PCI (7.0 [5.0–8.0] vs. 4.0 [2.0–6.0], respectively, *p* < 0.0001). The incidence of CV events, especially new coronary events, was not increased by the resumption of high‐intensity exercise (total cardiovascular events: 5.9% vs. 7.0%, *p* = 0.541; new coronary events: 1.5% vs. 2.9%, respectively, *p* = 0.250).

**TABLE 3:** Comparison between patients who performed high‐intensity exercise in the first year after PCI and the others.

	High‐intensity exercise in the first year (*n* = 205; 18%)	No high‐intensity exercise in the first year (*n* = 937; 82%)	*p*
Age at inclusion	59.5 [51.25–65.0]	61.0 [54.0–68.0]	0.018
Men	94.6%	87.4%	0.003
Diabetes mellitus at inclusion	6.4%	11.4%	0.037
Smoking at inclusion	18.7%	26.5%	0.021
Smoking 1‐year FU	4.4%	7.6%	0.096
Obesity at inclusion	9.8%	12.4%	0.291
Arterial hypertension at inclusion	34.5%	38.6%	0.277
Hypercholesterolemia at inclusion	39.9%	42.4%	0.524
None of those CV risk factor at inclusion	31.4%	23.9%	0.026
Emergency coronary angiography	27.3%	25.8%	0.660
LV wall motion abnormality at inclusion	39.4%	44.3%	0.211
LVEF at inclusion (%)	60.0 [50.0–65.75]	59.0 [50.0–61.0]	0.278
Complete revascularization	83.2%	77.8%	0.092
Number of stents per patient	1.0 [1.0–2.0]	1.0 [1.0–2.0]	0.103
Single‐vessel disease	76.0%	76.2%	0.944
Patient stented on LMCA	5.9%	1.7%	0.001
Patient stented on LAD	62.9%	60.3%	0.485
Patient stented on CX	22.9%	26.6%	0.280
Patient stented on RCA	36.1%	38.1%	0.592
Drug‐eluting stents	98.5%	99.6%	0.085
Duration of exercise before PCI (hours/week)	6.0 [5.0–9.0]	4.0 [3.0–6.0]	< 0.0001
Endurance exercise before PCI	86.4%	90.0%	0.147
Intensity of exercise before PCI			< 0.0001
Light‐intensity	3.4%	33.7%	
Moderate‐intensity	14.1%	52.0%	
Hight‐intensity	82.4%	14.3%	
Competition before PCI	30.6%	8.3%	< 0.0001
Duration of exercise after PCI (hours/week)	7.0 [5.0–8.0]	4.0 [2.0–6.0]	< 0.0001
Duration of exercise after PCI (only patient who resumed exercise, hours/week)	7.0 [5.0–8.0]	4.0 [3.0–7.0]	< 0.0001
Endurance exercise after PCI	90.7%	93.9%	0.109
Intensity of exercise after PCI			
No		10.4%	
Light‐intensity		39.7%	
Moderate‐intensity		49.9%	
Competition after PCI	23.4%	1.0%	< 0.0001
Change in intensity of exercise (post‐pre PCI)			< 0.0001
Decrease	0	35.2%	
No change	82.4%	60.6%	
Increase	17.6%	4.2%	
Time to resumption (months)	1.0 [1.0–3.0]	1.0 [1.0–3.0]	0.404
Total cardiovascular events	5.9% (*n* = 12)	7.0% (*n* = 66)	0.541
New coronary events	1.5% (*n* = 3)	2.9% (*n* = 27)	0.250
Atrial fibrillation	2.9% (*n* = 6)	2.2% (*n* = 21)	0.558
Ischemic stroke	0	0.4% (*n* = 4)	0.349
Ventricular arrhythmia	0	1.0% (*n* = 9)	0.159
Acute heart failure	1.5% (*n* = 3)	0.5% (*n* = 5)	0.148

Abbreviations: CV, cardio‐vascular; CX, circumflex coronary artery; FU, Follow‐up; LAD, left anterior descending coronary artery; LMCA, left main coronary artery; LV, left ventricular; LVEF, left ventricular ejection fraction; PCI, percutaneous coronary intervention; RCA, right coronary artery.

## 4 Discussion

In this prospective multicentre cohort of 1154 previously physically active patients who underwent PCI, resumption of exercise within the first year was common (91.6%). Cardiovascular outcomes were favorable, with no CV death and low incidence of recurrent coronary events (2.6%). Importantly, patients who resumed high‐intensity exercise did not experience a higher rate of adverse CV or coronary events, and none of the acute coronary syndromes occurred during high‐intensity exercise.

### 4.1 A Population With Rare Events During Follow‐Up

Compared with the general French CAD population described in the France PCI registry in 2022 [22], the percentages of patients with stent thrombosis or in‐stent restenosis were lower (0.4% vs. 0.9% and 0.3% vs. 1.5% respectively in our study vs. the France PCI registry). This could be related to the lower prevalence of CV risk factors in our cohort [23, 24], which was younger (61 vs. 69 years), with a lower prevalence of diabetes mellitus, obesity, and arterial hypertension (10.6% vs. 27.8%, 11.9% vs. 27.3%, and 37.8% vs. 59%, respectively); however, current smoking (25.1% vs. 25%) and dyslipidaemia (41.7% vs. 48.6%) rates were overall similar between both cohorts. Our population was highly active before their first event, with a training volume above the WHO recommendations [25].

This differs markedly from our earlier study published in 2019, which included CAD patients engaged in moderate‐to‐high‐intensity physical activity [17], where only 40% of the stents were drug‐eluting stents. The overall incidence of subsequent coronary events was around 16%, and stent thromboses occurred only in patients treated with non‐drug‐eluting stents. In contrast, in the present study, the widespread use of drug‐eluting stents shift appears to have a significant impact on reducing the recurrence of coronary events [26, 27]. Furthermore, regarding thrombosis, some studies also highlighted the importance of certain risk factors, such as advanced age, female gender, or the presence of small artery diseases [28, 29, 30], characteristics that were underrepresented in our population. Although the difference was not statistically significant, patients with recurrence had a higher prevalence of left main coronary artery (LMCA) stenting at index PCI. This trend is consistent with prior literature indicating that LMCA disease represents high‐risk coronary anatomy, with higher rates of ischemic events and repeat revascularization after PCI [31]. Accordingly, we cannot exclude that underlying anatomical risk contributed to recurrence in a subset of patients.

### 4.2 Resumption of Exercise

In our population, we observed a high rate of resumption of exercise (91.6%) in the year following PCI, regardless of the initial event. Only 18% of the patients resumed high‐intensity exercise (*n* = 205, 18%). These patients also engaged in more hours of exercise per week post‐PCI than the other patients and were therefore exposed to a greater cumulative physical activity load. In this sub‐group we did not observe more CV events, including coronary events. These patients were younger, with fewer baseline CV risk factors, but they were more likely to be male and there were no significant differences between groups as regards baseline echocardiographic or coronary angiographic characteristics.

Although the comparison may be underpowered due to the small number of patients with new coronary events, these individuals engaged in fewer hours of exercise. There was no significant difference in the intensity of exercise after PCI or in participation in competitive sports. However, patients in the recurrent coronary event group were more likely to have reduced the intensity of their exercise. Notably, none of the ACS occurred during high‐intensity exercise.

Our findings align with observational data indicating that maintaining or increasing physical activity after myocardial infarction is associated with lower mortality and recurrence. In the Corpus Christi Heart Project [1], patients who remained active or increased activity after a myocardial infarction had up to 78% lower risk of reinfarction and 89% lower risk of all‐cause mortality over 7 years. Our study extends this concept to patients treated with PCI. Moreover, mechanistic evidence from Munk et al. [27] indicates that supervised high‐intensity interval training over 6 months may reduce in‐stent late lumen loss, improve endothelial function, and lower inflammatory markers. Although that trial involved a small and selected sample under controlled conditions, it supports the concept that vigorous exercise is not only tolerable but may confer vascular benefit following PCI.

Together, these findings are consistent with the principles outlined in the 2020 European guidelines [15] and the 2025 North American clinical statement on sports cardiology [16], which indicate that, in the absence of symptoms, ventricular dysfunction, ischaemia or arrhythmias, previously active patients may return to more intense exercise or competitive sport 3–6 months after ACS or PCI, following appropriate evaluation. However, the European guidelines still consider resuming competitive activity within 12 months as a high‐risk feature for exercise‐induced adverse cardiac events, whereas the North American guidelines emphasize the importance of at least 2 years of statin therapy.

In this selected cohort of previously active individuals, resumption of high‐intensity exercise was not associated with a higher rate of early cardiovascular events. Our findings provide real‐world observational data but are not sufficient to conclude that high‐intensity exercise is safe for all patients after PCI. Further studies are needed to clarify which patients can safely engage in such activity.

### 4.3 Study Limitations

This study has certain undeniable limitations. This was not a randomized controlled trial; patients made the decision to resume exercise in accordance with their physician, potentially introducing a selection bias. The collection of physical activity data was based solely on self‐report, and wearables devices were not used to objectively assess exercise, making it difficult to precisely quantify the duration and intensity of exercise. While we have a fairly reliable understanding of the initial presence of CV risk factors and treatment, we do not know the levels of control of these factors in the year following PCI. Finally, the lack of statistical significance concerning several studied parameters appears due to the very low number of events during FU. A prospective study with a larger cohort of active individuals may help reveal some discriminating factors during the first year of follow‐up.

## 5 Perspective

In this large cohort of physically active patients who underwent PCI, we observed a very low rate of CV‐events during 1‐year follow‐up, including among those who resumed high‐intensity exercise. Importantly, no CV‐deaths occurred, and exercise‐triggered events were exceptionally rare. While these findings are encouraging, they primarily reflect outcomes in a younger, healthier, and highly active patient population and should not be generalized to all individuals undergoing PCI. Further studies with larger and more diverse cohorts are needed to better understand which patients can safely resume high‐intensity exercise and under which conditions.

## Author Contributions

J.M.G., F.S., L.C. contributed to the study design, subject recruitment, data acquisition, analyzed the data, and drafted the manuscript. S.C., S.D. contributed to the study design, subject recruitment, and data acquisition, revised the manuscript. S.G. contributed to the study design, data analysis, and revised the manuscript. B.G., S.A., F.C., C.H., F.I. contributed to the subject recruitment, revised the manuscript. All authors read and approved the final manuscript.

## Funding

This work was funded by the “Club des Cardiologues du Sport”.

## Ethics Statement

The study was approved by the Dijon Committee for the Protection of Persons (approval no. 2018‐A01169‐46) and received a compliance agreement from the French National Commission on Informatics and Liberties (CNIL) on April 26, 2018 (no. 2177339 v0).

## Consent

All participants provided informed consent.

## Conflicts of Interest

F.S. reported consulting fees from Bristol‐Myers Squibb, AbbVie, Servier, and Sanofi outside the submitted work. F.I. reported consulting fees from Novo Nordisk and Sanofi outside the submitted work.

## Data Availability

The data underlying this article will be shared on reasonable request to the corresponding author.

## References

[1] L. Steffen‐Batey , M. Z. Nichaman , D. C. G. Jr , et al., “Change in Level of Physical Activity and Risk of All‐Cause Mortality or Reinfarction,” Circulation 102 (2000): 2204–2209, 10.1161/01.cir.102.18.2204. 11056093

[2] H. Domínguez , C. Torp‐Pedersen , L. Koeber , and C. Rask‐Madsen , “Prognostic Value of Exercise Testing in a Cohort of Patients Followed for 15 Years After Acute Myocardial Infarction,” European Heart Journal 22 (2001): 300–306, 10.1053/euhj.2000.2281. 11161948

[3] R. A. H. Stewart , C. Held , N. Hadziosmanovic , et al., “Physical Activity and Mortality in Patients With Stable Coronary Heart Disease,” Journal of the American College of Cardiology 70 (2017): 1689–1700, 10.1016/j.jacc.2017.08.017. 28958324

[4] M. Lahtinen , T. Toukola , M. J. Junttila , et al., “Effect of Changes in Physical Activity on Risk for Cardiac Death in Patients With Coronary Artery Disease,” American Journal of Cardiology 121 (2018): 143–148, 10.1016/j.amjcard.2017.10.002. 29126583

[5] C. Vrints , F. Andreotti , K. C. Koskinas , et al., “2024 ESC Guidelines for the Management of Chronic Coronary Syndromes,” European Heart Journal 45 (2024): 3415–3537, 10.1093/eurheartj/ehae177. 39210710

[6] B. Gerardin , P. Guedeney , A. Bellemain‐Appaix , et al., “Life‐Threatening and Major Cardiac Events During Long‐Distance Races: Updates From the Prospective RACE PARIS Registry With a Systematic Review and Meta‐Analysis,” European Journal of Preventive Cardiology 28 (2020): 679–686, 10.1177/2047487320943001. 34021577

[7] B. Gerardin , J.‐P. Collet , H. Mustafic , et al., “Registry on Acute Cardiovascular Events During Endurance Running Races: The Prospective RACE Paris Registry,” European Heart Journal 37 (2016): 2531–2541, 10.1093/eurheartj/ehv675. 26715168

[8] P. Bohm , J. Scharhag , and T. Meyer , “Data From a Nationwide Registry on Sports‐Related Sudden Cardiac Deaths in Germany,” European Journal of Preventive Cardiology 23 (2016): 649–656, 10.1177/2047487315594087. 26130495 PMC4776219

[9] G. Claessen , T. M. H. Eijsvogels , C. M. Albert , et al., “Coronary Atherosclerosis in Athletes: Emerging Concepts and Preventive Strategies,” European Heart Journal 46 (2025): ehae927, 10.1093/eurheartj/ehae927. PMC11887545 39791533

[10] R. D. Bosscher , C. Dausin , P. Claus , et al., “Lifelong Endurance Exercise and Its Relation With Coronary Atherosclerosis,” European Heart Journal 44 (2023): 2388–2399, 10.1093/eurheartj/ehad152. 36881712 PMC10327878

[11] V. L. Aengevaeren , A. Mosterd , E. A. Bakker , et al., “Exercise Volume Versus Intensity and the Progression of Coronary Atherosclerosis in Middle‐Aged and Older Athletes: Findings From the MARC‐2 Study,” Circulation 147 (2023): 993–1003, 10.1161/circulationaha.122.061173. 36597865

[12] C. J. Womack , P. R. Nagelkirk , and A. M. Coughlin , “Exercise‐Induced Changes in Coagulation and Fibrinolysis in Healthy Populations and Patients With Cardiovascular Disease,” Sports Medicine 33 (2003): 795–807, 10.2165/00007256-200333110-00002. 12959620

[13] S. Brunner , K. Rizas , W. Hamm , M. Mehr , and K. Lackermair , “Effect of Physical Exercise on Platelet Reactivity in Patients With Dual Antiplatelet Therapy,” International Journal of Sports Medicine 39 (2018): 646–652, 10.1055/a-0631-3302. 29902806

[14] S. Sharma , A. Merghani , and L. Mont , “Exercise and the Heart: The Good, the Bad, and the Ugly,” European Heart Journal 36 (2015): 1445–1453, 10.1093/eurheartj/ehv090. 25839670

[15] A. Pelliccia , S. Sharma , S. Gati , et al., “2020 ESC Guidelines on Sports Cardiology and Exercise in Patients With Cardiovascular Disease,” European Heart Journal 42 (2020): 17–96, 10.1093/eurheartj/ehaa605. 32860412

[16] J. H. Kim , A. L. Baggish , B. D. Levine , et al., “Clinical Considerations for Competitive Sports Participation for Athletes With Cardiovascular Abnormalities A Scientific Statement From the American Heart Association and American College of Cardiology,” Journal of the American College of Cardiology 85 (2025): 1059–1108, 10.1016/j.jacc.2024.12.025. 39976316 PMC12145891

[17] J.‐M. Guy , M. Wilson , F. Schnell , et al., “Incidence of Major Adverse Cardiac Events in Men Wishing to Continue Competitive Sport Following Percutaneous Coronary Intervention,” Archives of Cardiovascular Diseases 112 (2019): 226–233, 10.1016/j.acvd.2018.11.008. 30612894

[18] G. A. Borg , “Psychophysical Bases of Perceived Exertion,” Medicine and Science in Sports and Exercise 14 (1982): 377, 10.1249/00005768-198205000-00012. 7154893

[19] R. Persinger , C. Foster , M. Gibson , D. C. W. Fater , and J. P. Porcari , “Consistency of the Talk Test for Exercise Prescription,” Medicine and Science in Sports and Exercise 36 (2004): 1632–1636. 15354048

[20] D. Bok , M. Rakovac , and C. Foster , “An Examination and Critique of Subjective Methods to Determine Exercise Intensity: The Talk Test, Feeling Scale, and Rating of Perceived Exertion,” Sports Medicine 52 (2022): 2085–2109, 10.1007/s40279-022-01690-3. 35507232

[21] L. Vanhees , B. Rauch , M. Piepoli , et al., “Importance of Characteristics and Modalities of Physical Activity and Exercise in the Management of Cardiovascular Health in Individuals With Cardiovascular Disease (Part III),” European Journal of Preventive Cardiology 19 (2012): 1333–1356, 10.1177/2047487312437063. 22637740

[22] France PCI Registry 2022 Annual Report , “French National Interventional Cardiology Registry,” https://www.francepci.com.

[23] D. L. Steen , I. Khan , K. Andrade , A. Koumas , and R. P. Giugliano , “Event Rates and Risk Factors for Recurrent Cardiovascular Events and Mortality in a Contemporary Post Acute Coronary Syndrome Population Representing 239 234 Patients During 2005 to 2018 in the United States,” J Am Hear Assoc: Cardiovasc Cerebrovasc Dis 11 (2021): e022198, 10.1161/jaha.121.022198. PMC9238606 35475346

[24] C. Magnussen , F. M. Ojeda , D. P. Leong , et al., “Global Effect of Modifiable Risk Factors on Cardiovascular Disease and Mortality,” New England Journal of Medicine 389 (2023): 1273–1285, 10.1056/nejmoa2206916. 37632466 PMC10589462

[25] F. C. Bull , S. S. Al‐Ansari , S. Biddle , et al., “World Health Organization 2020 Guidelines on Physical Activity and Sedentary Behaviour,” British Journal of Sports Medicine 54 (2020): 1451–1462, 10.1136/bjsports-2020-102955. 33239350 PMC7719906

[26] M. J. Zellweger , C. Kaiser , R. Jeger , et al., “Coronary Artery Disease Progression Late After Successful Stent Implantation,” Journal of the American College of Cardiology 59 (2012): 793–799, 10.1016/j.jacc.2011.11.024. 22361397

[27] P. S. Munk , E. M. Staal , N. Butt , K. Isaksen , and A. I. Larsen , “High‐Intensity Interval Training May Reduce In‐Stent Restenosis Following Percutaneous Coronary Intervention With Stent Implantation A Randomized Controlled Trial Evaluating the Relationship to Endothelial Function and Inflammation,” American Heart Journal 158 (2009): 734–741, 10.1016/j.ahj.2009.08.021. 19853690

[28] F. Philip , S. Stewart , and J. A. Southard , “Very Late Stent Thrombosis With Second Generation Drug Eluting Stents Compared to Bare Metal Stents: Network Meta‐Analysis of Randomized Primary Percutaneous Coronary Intervention Trials,” Catheterization and Cardiovascular Interventions 88 (2016): 38–48, 10.1002/ccd.26458. 26916633

[29] A. Polimeni , S. Sorrentino , C. Spaccarotella , et al., “Stent Thrombosis After Percutaneous Coronary Intervention From Bare‐Metal to the Last Generation of Drug‐Eluting Stents,” Interventional Cardiology Clinics 11 (2022): 465–473, 10.1016/j.iccl.2022.07.002. 36243491

[30] F. Condello , C. Spaccarotella , S. Sorrentino , C. Indolfi , G. G. Stefanini , and A. Polimeni , “Stent Thrombosis and Restenosis With Contemporary Drug‐Eluting Stents: Predictors and Current Evidence,” Journal of Clinical Medicine 12 (2023): 1238, 10.3390/jcm12031238. 36769886 PMC9917386

[31] J. M. Stolker , D. J. Cohen , K. F. Kennedy , et al., “Repeat Revascularization After Contemporary Percutaneous Coronary Intervention: An Evaluation of Staged, Target Lesion, and Other Unplanned Revascularization Procedures During the First Year,” Circulation. Cardiovascular Interventions 5 (2012): 772–782, 10.1161/circinterventions.111.967802. 23093553

